# LazyAF, a pipeline for accessible medium-scale in silico prediction of protein-protein interactions

**DOI:** 10.1099/mic.0.001473

**Published:** 2024-07-05

**Authors:** Thomas C. McLean

**Affiliations:** 1 Department of Molecular Microbiology, John Innes Centre, Norwich, UK

**Keywords:** alphafold, colabfold, modelling, protein-protein interactions, structure prediction

## Abstract

Artificial intelligence has revolutionized the field of protein structure prediction. However, with more powerful and complex software being developed, it is accessibility and ease of use rather than capability that is quickly becoming a limiting factor to end users. LazyAF is a Google Colaboratory-based pipeline which integrates the existing ColabFold BATCH software to streamline the process of medium-scale protein-protein interaction prediction. LazyAF was used to predict the interactome of the 76 proteins encoded on the broad-host-range multi-drug resistance plasmid RK2, demonstrating the ease and accessibility the pipeline provides.

## Availability

LazyAF is freely available at https://github.com/ThomasCMcLean/LazyAF.

## Introduction

The integration of artificial intelligence-based protein structure prediction software, such as AlphaFold2 and RoseTTAFold, into hosted notebook services such as Google Colaboratory has dramatically improved accessibility [1,3]. These platforms are not only user-friendly but also provide access to powerful graphics processing units (GPU) to accelerate predictions of large or complex protein structures, thereby removing the requirement for local high-performance computing clusters that are not widely available or require advanced computer literacy to use.

As the speed and accessibility of protein structure prediction increase so does the potential scale, ranging from single protein structure predictions to screening large datasets of proteins for new protein–protein interactions (PPI). This work details LazyAF, a Google Colaboratory-based pipeline designed to work with ColabFold BATCH [3] to streamline the process of screening large datasets of proteins for novel PPIs and to make it accessible to users with less bioinformatics experience. The medium-throughput power of LazyAF makes it well-suited for use in the field of microbiology particularly for the investigation of PPIs in plasmids, virulence islands and viruses. These microbial elements encode for more manageable numbers of proteins in comparison to entire eukaryotic genomes. This manuscript demonstrates the accessibility of this pipeline and its ability to perform automatic medium-throughput prediction of protein complexes in an all-versus-all scenario using the 76 proteins encoded on a multidrug-resistance plasmid RK2 as an example [4]. LazyAF is designed to lower the entry barrier for medium-scale PPI prediction and empower more wet-bench researchers to integrate *in silico* modelling into their workflow.

## Software description

**Fig. 1.**
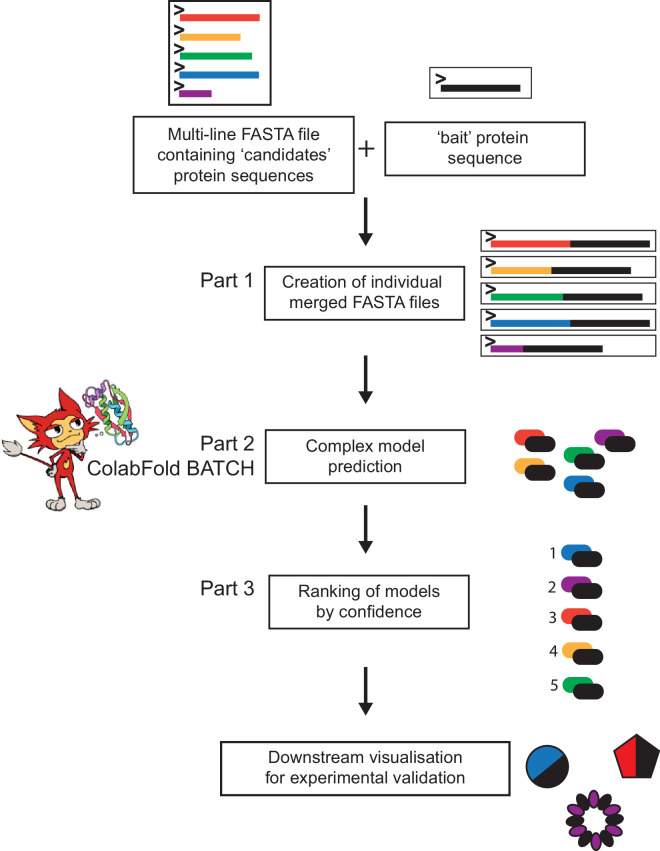
The LazyAF pipeline. LazyAF Part 1 concatenates a FASTA file containing multiple ‘candidate’ protein sequences with a single ‘bait’ protein sequence and outputs each as an individual FASTA file. This collection of individual FASTA files is then used as the input for AlphaFold2-Multimer-based protein structure prediction using ColabFold BATCH. This takes advantage of powerful cloud-based GPUs to enable rapid modelling without the need of local high-performance computing facilities. LazyAF Part 3 retrieves the results from the modelling in Part 2 and ranks the likelihood of protein-protein interactions by their ranking confidence score. These results can be used to generate new hypotheses but should ideally be experimentally validated.

AlphaFold2-Multimer-based prediction of PPIs is conceptually similar to a co-immunoprecipitation experiment where a protein of interest is used as bait to pull down its interacting protein partners from the total cell lysate. LazyAF takes a protein as a ‘bait’ and a list of other proteins as ‘candidates’ to automatically run an AlphaFold2-Multimer prediction between the bait and each candidate before ranking the likelihood of their interactions. LazyAF consists of two open-source Google Colaboratory notebooks (see data availability). At the core of LazyAF is ColabFold BATCH which is also available via Google Colaboratory (https://github.com/sokrypton/ColabFold) [3,5]. An outline of LazyAF is depicted in Fig. 1. A step-by-step protocol can be found in Note S1 (available in the online version of this article).

### Part 1 – preparation of input files for ColabFold BATCH

Input for LazyAF Part 1 consists of (i) a name and sequence of a ‘bait’ protein and (ii) a multi-line FASTA file containing sequences of ‘candidate’ proteins. It is recommended to download the ‘candidate’ sequence file from NCBI (see Note S1) as this file contains a unique identifier for each candidate protein sequence.

The output of LazyAF Part 1 is a collection of individual FASTA files each with the sequence of the ‘bait’ and that of a ‘candidate’ joined via a colon. A colon separator designates separate chains for the subsequent AlphaFold2-Multimer-based protein co-folding. Each FASTA file is uniquely named based on the name of the ‘bait’ and the unique identifier of each ‘candidate’ protein. All individual FASTA files are stored in a cloud-based Google Drive folder.

### Part 2 – structure prediction in batch mode by ColabFold BATCH

The output folder from LazyAF Part 1 can be used directly as the input for ColabFold BATCH. For further information and methodology on ColabFold BATCH see [3].

### Part 3 – ranking likelihood of bait-candidate interactions

LazyAF Part 3 simplifies the collation and analysis of the numerous PPI predictions from ColabFold BATCH [3]. With each prediction producing five models alongside other accessory files, the amount of data is large and challenging to analyse manually. LazyAF Part 3 locates the JSON file associated with the top-ranked prediction for each co-folding and copies them into a new folder for subsequent analysis. The predicted template modelling score (pTM) and predicted interface template modelling score (ipTM) are then extracted from these JSON files, and a ranking score (ranking confidence) is calculated for each PPI using the following formula (ranking confidence=0.2 pTM + 0.8 ipTM) [6]. These ranking confidence scores can then be sorted from high to low to assess the quality of models and the likelihood of interaction between each bait-candidate protein pair.

### LazyAF predicts the potential interactome of 76 proteins encoded on the RK2 plasmid

**Fig. 2.**
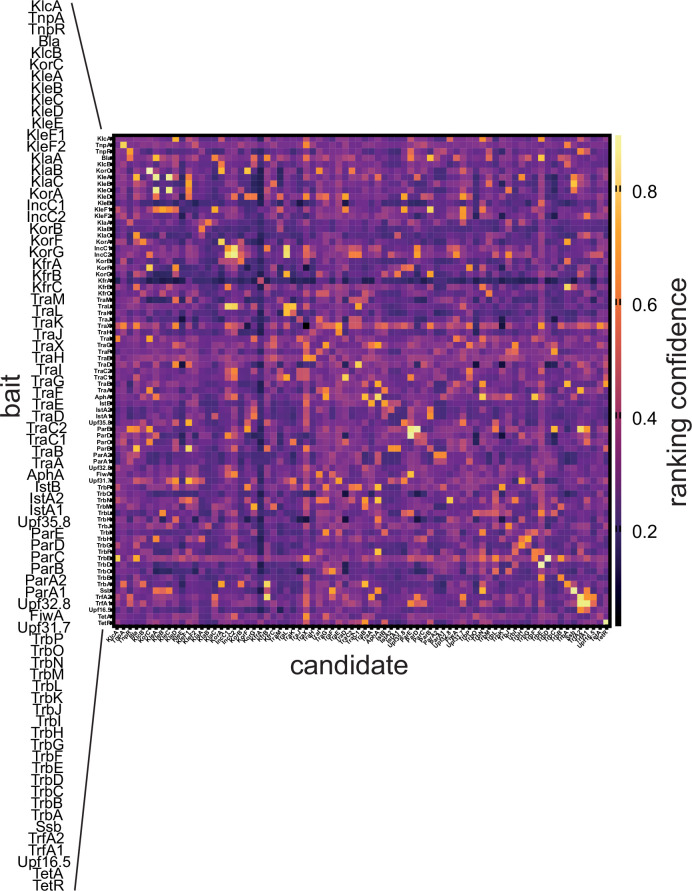
Heatmap displaying ranking confidence scores for 5776 interactions from the 76 proteins encoded on the RK2 plasmid. Proteins are ordered by the sequential genomic locations of their encoding genes on RK2. The ranking confidence score is calculated using the formula: ranking confidence score=0.2 pTM + 0.8 ipTM for the top ranked model of each protein-protein pair prediction. The colour scale is a gradient from bright yellow (high ranking confidence score) down to deep purple (low ranking confidence score). Predicted protein-protein interactions directly along the diagonal axis could be indicative of homodimerization. Predicted protein-protein interactions extending from the diagonal axis could be considered indicative of operonal interactions.

**Fig. 3.**
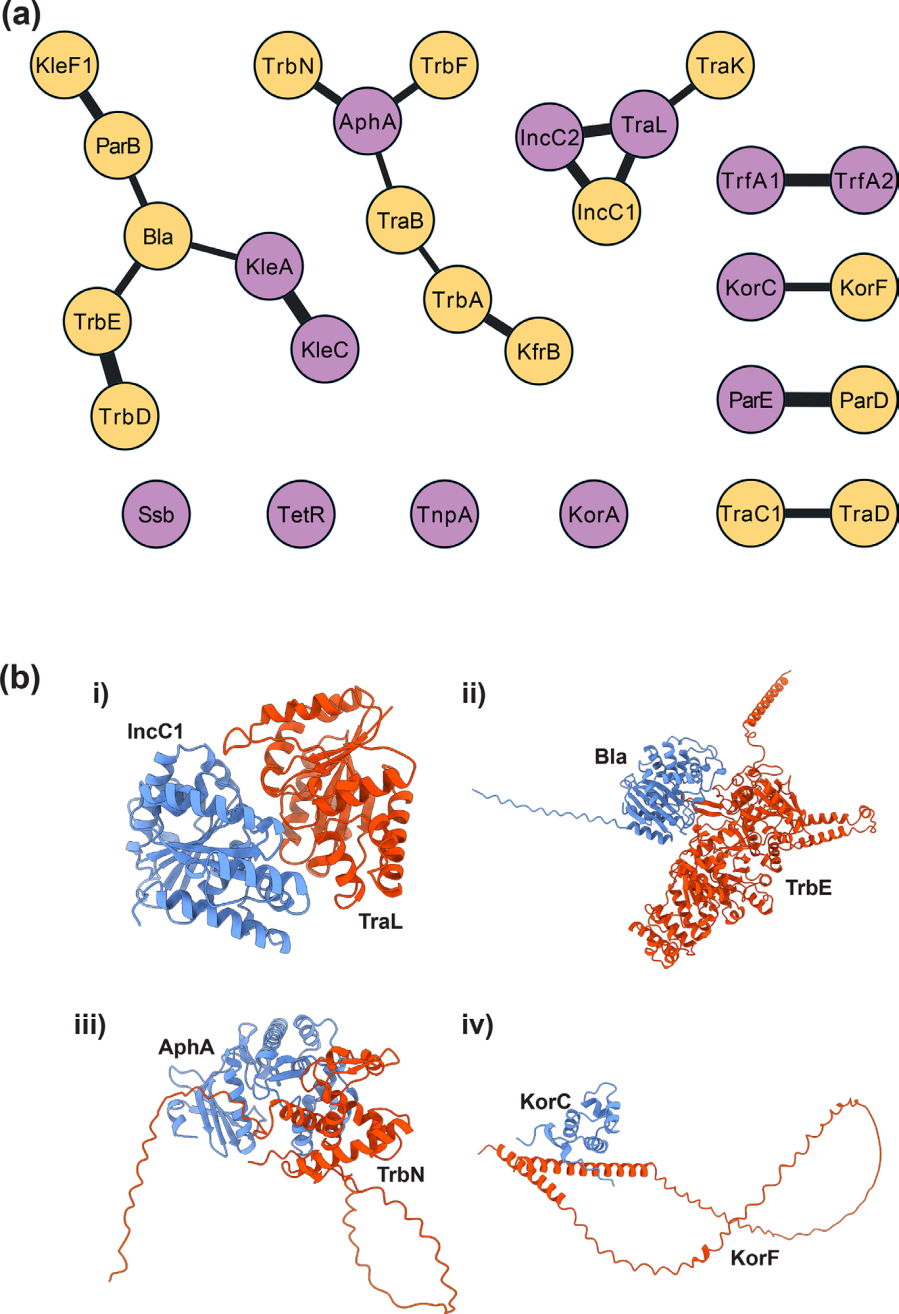
**(a**) An interaction network for most likely protein-protein interaction pairs from the all-versus-all screen of 5776 potential interactions from the 76 proteins encoded on the RK2 plasmid. Yellow nodes indicate proteins predicted to not self-interact. Purple nodes indicate proteins predicted to self-interact. Nodes are included if their ranking confidence scores are >0.7 in both primary and reciprocal pairings. The thickness of the line is proportional to a maximal ranking confidence score. (**b**) Predicted models of four high likelihood PPIs predicted using LazyAF: (i) IncC1 (blue) : TraL (red); (ii) Bla (blue) : TrbE (red); (iii) AphA (blue) : TrbN (red); (iv) KorC (blue) : KorF (red).

To demonstrate the accessibility of LazyAF, and its ability to perform automatic medium-throughput prediction of protein complexes, LazyAF was applied to an all-versus-all scenario using the 76 proteins encoded on a multi-drug resistance plasmid RK2. A multi-line FASTA file containing all protein sequences encoded on the RK2 genome (BN000925.1) was retrieved from NCBI and uploaded to Google Drive. Each protein sequence (76 in total) was used as a bait sequence in LazyAF Part 1 to generate 76×76, i.e. 5776 FASTA files with every possible pairwise combination of protein-protein interactions. This was achieved with 76 singular runs using different bait sequences each time. In its current form LazyAF is unable to automate this further however the generation of concatenated fastas in LazyAF Part 1 is rapid and changing each bait sequence is not manually intensive. For larger all-versus-all screens it would be advisable to trim the starting database to a subset of relevant proteins. This folder was then used as the input directory for ColabFold v1.5.2 AlphaFold2 MMseqs2 BATCH with the following settings: msa_mode: MMseqs2 (UniRef +Environmental), num_models: 5, num_recycles: 3, stop_at_score: 100. The notebook was run on High-RAM A100 or V100 GPUs depending on their availability on Google Colaboratory. The resulting folder containing the outputs from AlphaFold2 predictions was used as the input directory for LazyAF Part 3 which copied the top-ranked JSON files for each predicted PPI into an analysis folder. Part 3 also retrieved the pTM and ipTM scores for each top-ranked model and calculated the ranking confidence score (0.2pTM +0.8 ipTM). These values were stored in an output CSV file on Google Drive (Table S1). A heatmap of ranking confidence scores for 5776 PPIs was plotted using GraphPad Prism 10 (Fig. 2), and an interaction network was generated using Cytoscape with default parameters (Fig. 3a).

It was noted that the heatmap of ranking confidence scores is not symmetrical (Fig. 2), suggesting that a score of co-folding between protein A (bait):protein B (candidate) sometimes is different from that of a co-folding between protein B (bait):protein A (candidate). Whilst most variation in ranking confidence was minimal (< 0.1) some larger changes were observed. The KlaC-KleF1 PPI exhibited the most extreme change with the ranking confidence score dropping over 75 % from 0.712 to 0.164 when the bait and candidate roles were switched. Variation is to be expected due to the nature of model building by iterative refinement, however to reduce the likelihood of false positives only the PPIs with a ranking confidence score of >0.7 in both directions were considered [7,8]. Whilst this threshold is lower than the 0.8 ranking confidence threshold originally used by Wallner *et al*. [6] this is balanced by the stringency added through the requirement of the ranking confidence being >0.7 in both directions. This threshold should be modified to fit the experimental aims and used in collaboration with other methods used to determine PPIs of interest for further investigation. Among 5776 predictions, 50 predicted PPIs have ranking confidence scores>0.7 in a single direction (i.e. protein A [bait]:protein B [candidate]) and also in the reciprocal direction (i.e. protein B [bait]:protein A [candidate]). These 50 predicted PPIs were considered to be of high likelihood (Fig. 3a). Several of the top predicted PPIs have been experimentally validated previously, particularly the homodimerization of transcriptional regulators KorA [9], TetR [10], and the transposase TnpA [11]. This screen also suggested many new potential protein complexes (Table S1). To highlight the diversity of interactions, the predicted models of four of these high likelihood novel interactions (IncC1:TraL; Bla:TrbE; AphA:TrbN; KorC:KorF) are presented in Fig. 3b.

Other PPI databases, such as STRING, provide complementary methods of PPI analysis to LazyAF-derived Cytoscape networks. STRING integrates a wide range of experimental and correlative genomic data, however plasmids are not currently searchable elements on the database [12]. In an attempt to contrast STRING with the LazyAF-derived network a custom database (STRG0A83CND) was created using the RK2 coding sequences appended to an *Escherichia coli* AB1157 genome. STRING found evidence suggesting a functional link for just a single interaction from those displayed in Fig. 3a: between ParD and ParE with a combined score of 0.418 out of a maximum score of 1 [12]. Any PPIs predicted by LazyAF, STRING or other software should be experimentally validated and characterized further. However this highlights the potential of LazyAF-derived networking as a broad hypothesis-generating tool.

## Conclusion

LazyAF provides a highly accessible pipeline that can be used in any web browser and can utilize powerful cloud-based hardware to facilitate predictions. Due diligence should always be taken to manually examine models, checking other parameters such as the per-residue confidence (pLDDT), the pDockQ/mpDockQ scores [13, 14], and validation by complementary *in vitro*/*in vivo* methodologies. Alternative software, such as AlphaPulldown [15], exists and goes well beyond the capabilities of LazyAF. However, this Python-based package requires substantial bioinformatic expertise to install and execute. LazyAF provides a slimmed-down, accessible pipeline for *in silico* pulldown experiments for users with less bioinformatics experience. As the line blurs between traditional wet and dry laboratory research, the anticipation is that LazyAF will be of most use to wet-lab scientists who are keen to integrate *in silico* protein structure predictions into their workflow.

## supplementary material

10.1099/mic.0.001473 Uncited Supplementary Material 1.

## Abbreviations

GPU: graphics processing unit
ipTM: predicted interface template modelling score
pLDDT: predicted local distance difference test
PPI: protein-protein interaction
pTM: predicted templated modelling score
