# Supplementary material for: LazyAF, a pipeline for accessible medium-scale in silico prediction of protein-protein interactions
Source: Microbiology (Reading). 2024 Jul 5;170(7):001473. doi: 10.1099/mic.0.001473 (PMC11316561; doi:10.1099/mic.0.001473)
Supplement: Uncited Supplementary Material 1. [file mic-170-01473-s001.pdf]

SUPPLEMENTARY INFORMATION

Supplementary Table 1. Output from LazyAF Part 3 for PPIs with ranking confidence scores > 0.7.

Candidate	Bait	pTM	ipTM	Ranking_confidence
KorC	KorC	0.91	0.89	0.894
TrbE	TrbD	0.85	0.89	0.882
TrbD	TrbE	0.85	0.89	0.882
ParE	ParD	0.77	0.9	0.874
ParD	ParE	0.76	0.9	0.872
Ssb	Ssb	0.87	0.87	0.87
TetR	TetR	0.87	0.87	0.87
KleA	KleA	0.86	0.86	0.86
KleA	KleC	0.86	0.86	0.86
IncC2	IncC2	0.9	0.84	0.852
KleC	KleA	0.86	0.85	0.852
TrfA1	TrfA1	0.78	0.86	0.844
TrfA1	TrfA2	0.78	0.86	0.844
KleC	KleC	0.85	0.84	0.842
ParE	ParE	0.88	0.83	0.84
TrfA2	TrfA2	0.86	0.82	0.828
IncC2	TraL	0.88	0.81	0.824
IncC2	IncC1	0.77	0.83	0.818

IncC1	IncC2	0.77	0.83	0.818
TrfA2	KfrB	0.88	0.8	0.816
ParE	TrfA2	0.87	0.8	0.814
AphA	AphA	0.85	0.8	0.81
ParB	KleF1	0.81	0.81	0.81
TraL	IncC1	0.76	0.82	0.808
IncC1	TraL	0.76	0.82	0.808
TrbA	KfrB	0.82	0.8	0.804
KfrB	TrbA	0.82	0.8	0.804
KleF1	ParB	0.81	0.8	0.802
TraL	IncC2	0.86	0.78	0.796
KorA	KorA	0.78	0.8	0.796
TnpA	TnpA	0.82	0.79	0.796
TraC1	TraD	0.72	0.81	0.792
TrbE	KlcA	0.79	0.79	0.79
TraD	TraC1	0.71	0.81	0.79
KorF	KorC	0.47	0.86	0.782
AphA	TrbN	0.74	0.79	0.78
TrbN	Upf32.8	0.71	0.79	0.774
Bla	TrbE	0.8	0.76	0.768
KorC	KorF	0.47	0.84	0.766
AphA	TrbF	0.71	0.78	0.766
TrbN	AphA	0.74	0.77	0.764

KleB	Ssb	0.81	0.75	0.762
TraL	TraK	0.69	0.78	0.762
ParB	Bla	0.84	0.74	0.76
TrbF	AphA	0.71	0.77	0.758
FiwA	ParE	0.78	0.75	0.756
Bla	ParB	0.84	0.73	0.752
TraK	TraL	0.67	0.77	0.75
TrbP	AphA	0.81	0.73	0.746
Bla	KleA	0.85	0.72	0.746
TraL	TraL	0.84	0.72	0.744
AphA	TraB	0.79	0.73	0.742
Upf31.7	KleF1	0.74	0.74	0.74
KleA	Bla	0.85	0.71	0.738
ParE	TrbC	0.53	0.79	0.738
KorC	KorA	0.72	0.74	0.736
TrbA	TraB	0.76	0.73	0.736
TraB	TrbA	0.76	0.73	0.736
Ssb	TrbN	0.68	0.75	0.736
TraA	TrfA2	0.84	0.7	0.728
KlcA	KleD	0.79	0.71	0.726
KorG	TraL	0.74	0.72	0.724
Upf35.8	KleF1	0.68	0.73	0.72
KleF1	KlaC	0.56	0.75	0.712

TrbM	KorG	0.66	0.72	0.708
Upf31.7	TrbH	0.74	0.7	0.708
TraB	AphA	0.77	0.69	0.706
TrbE	Bla	0.77	0.69	0.706
TraX	TraE	0.81	0.68	0.706
TraI	KorC	0.56	0.74	0.704
IstB	TraA	0.76	0.69	0.704
TrbH	TrbG	0.63	0.72	0.702

Supplementary Note 1. A step-by-step protocol on how to run LazyAF

1. Download your genome of interest from NCBI as coding sequences in the FASTA Protein format (see picture below). This will give you a file named *sequence.txt* containing a list of FASTAs for every coding sequence identified within the genome of interest. This will be the 'candidate' proteins file. I suggest renaming this *sequence.txt* file to include the genome name.

Note: alternatively, you can curate your version of the 'candidate' protein file by manually pasting protein sequences in FASTA format into a text file.

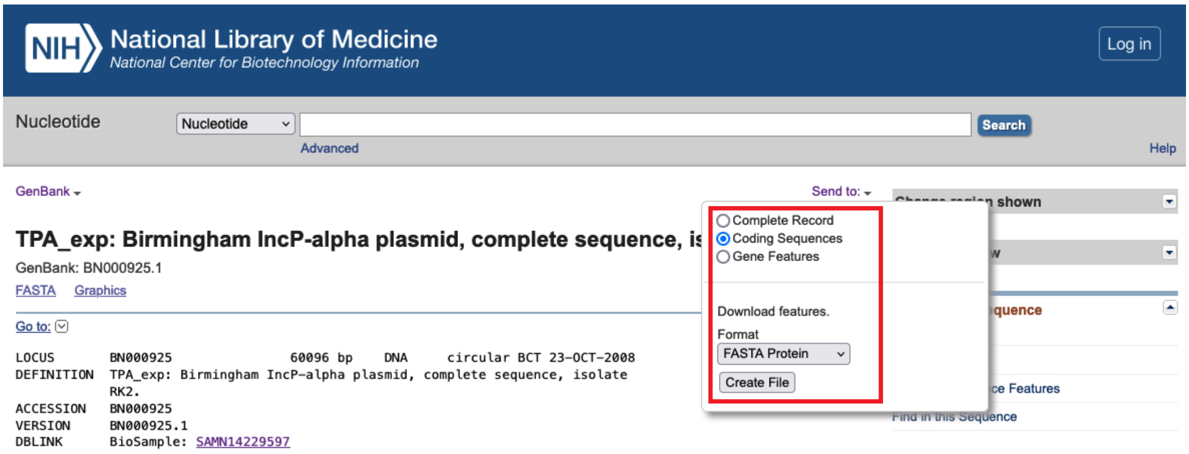

2. Create a folder in Google Drive in the *My Drive* section with the name *input* (see picture below). Upload your *sequence.txt* file from Step 1 to the *input* folder.

Note: you will need to register for a Google Drive account if you do not already have one.

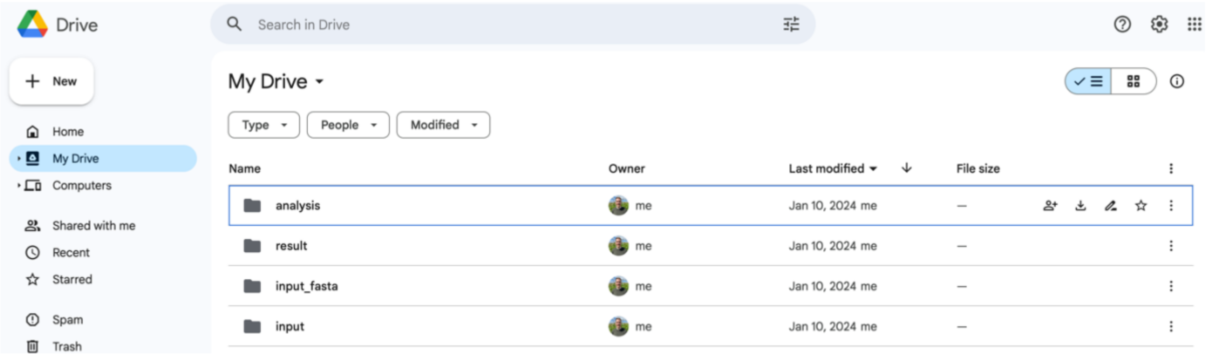

3. Open LazyAF Part 1 in your web browser (see picture below). If you have renamed any folders or files make sure these match up to the *input_dir, result_dir* and *input_file.* Then provide the bait protein sequence and name while making sure to remove any non-amino acid letters, symbols, or spaces (a common one can be a terminal *) from the protein sequence. When ready click *runtime -> run all* to run the script.

Note: connecting to your Google Drive is required. The time required to complete the process depends on the number of coding sequences in the 'candidate' protein file. You can follow the process in the Google Drive input_fasta folder but on average a few minutes should suffice to complete this step.

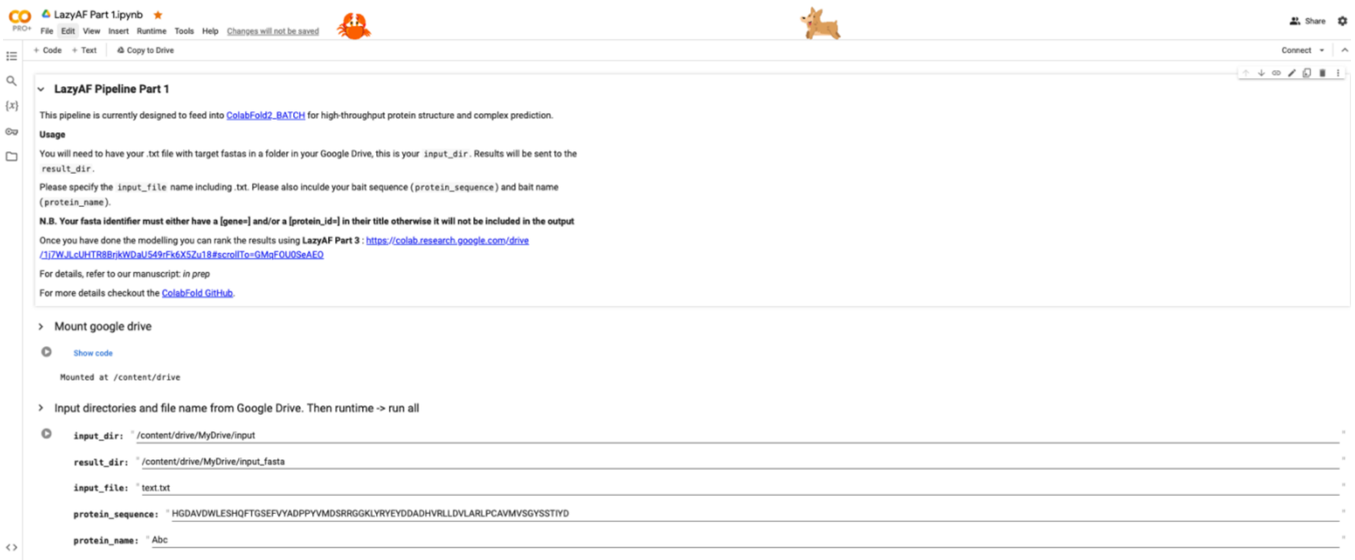

4. Once complete proceed to LazyAF Part 2 (ColabFold v1.5.5: AlphaFold2 w/ MMseqs2 BATCH at time of publication) and follow the detailed instructions provided. If you have not modified folder names then the default should match the ones in your Google Drive (N.B. there is no need to create the *result_dir* folder, the software creates it as required). For new users, it is recommended to use the default settings. If you have access to the Pro or Pro+ plans, you should change your GPU to either the preferable A100 or the V100 if the A100 is not available. This can be done by clicking *runtime -> change runtime type -> select A100 or V100*. When ready, click *runtime -> run all* to run the script.

Note: connecting to your Google Drive is required. The output from this modeling step can require large storage space on your Google Drive, it is recommended to upgrade your Google Drive plan to 100 Gb of storage capacity. It is also recommended to upgrade your Google Colab plan to Pro+. The upgrade not only gives users access to the more powerful hardware i.e. A100 GPU but also allows running of the notebook in the background. The compute unit requirements and time for complete modeling depend on the number of protein-protein interactions and the size of each protein. As a rule of thumb, if you are using an A100 GPU you will use ~15-20 compute units an hour and might model between 5-20 interactions per hour. Make sure to keep your compute units (drop-down arrow in the top right of the screen -> view resources) topped up to prevent disconnection from your runtime. ColabFold v1.5.5: AlphaFold2 w/ MMseqs2 BATCH tracks its own progress. If ColabFold is disconnected for any reason or crashes, click runtime -> disconnect and delete runtime, refresh your browser page, and restart the process. ColabFold BATCH will pick up where it left off.

5. Once all modeling is complete proceed to LazyAF Part 3. Make sure all the directories are named correctly and change the name of the output analysis file *csv_name* if you wish. Unless you have modified the naming scheme of the input files, then leave the fields *split_1* and *split_2* as default. When ready, click *runtime -> run all* to run the script.

Note: connecting to your Google Drive is required. The time required to complete the process depends on the number of predictions. You can follow the process in the Google Drive analysis folder but on average a few minutes should suffice for most jobs. When complete the folder should contain both the top-ranked JSON files for each model and the output analysis CSV file containing columns for the bait and candidate proteins, pTM, ipTM, and ranking_confidence score.
